# Robotic, laparoscopic, and open nephrectomy and 30-day pulmonary morbidity: a procedure-stratified cohort study with intraoperative ventilation data

**DOI:** 10.1007/s11701-026-03779-7

**Published:** 2026-08-11

**Authors:** Husny Mahmud, Amir Zabida, Ido Amit, Yoram Mor, Haim Berkenstadt, Dina Orkin, Dorit E. Zilberman, Barak Rosenzweig, Orith Portnoy, Menachem Laufer, Zohar A. Dotan

**Affiliations:** 1 Faculty of Medical and Health Sciences, Tel Aviv University, Tel Aviv, Israel; 2 Department of Urology, Sheba Medical Center, Sheba Road 1, Ramat Gan, 5262000 Israel; 3 Department of Anesthesiology, Sheba Medical Center, Ramat Gan, Israel; 4 Division of Operating Rooms, Sheba Medical Center, Ramat Gan, Israel; 5 Department of Diagnostic Radiology, Sheba Medical Center, Ramat Gan, Israel

**Keywords:** Nephrectomy, Robotic surgery, Postoperative pulmonary complications, Laparoscopy, Perioperative outcomes

## Abstract

Previous studies of heterogeneous abdominal procedures have reported higher pulmonary-complication rates after robot-assisted surgery, but whether this applies to nephrectomy is uncertain. We evaluated surgical approach and 30-day postoperative pulmonary complications (PPCs) after partial and radical nephrectomy. We studied 1,503 adults undergoing robotic, laparoscopic, or open nephrectomy from 2008 to 2025. Procedure-stratified logistic regression assessed a prespecified PPC composite. The partial-nephrectomy model included tumor complexity and clinical covariates. Sparse-data and additional sensitivity analyses were performed. Minute-level ventilation data were analyzed descriptively. Overall PPC incidence was 4.5% (68/1,503). In partial nephrectomy, robotic surgery was associated with lower adjusted PPC odds than laparoscopy (adjusted odds ratio [aOR], 0.26; 95% CI, 0.08–0.87) and open surgery (aOR, 0.18; 95% CI, 0.07–0.43). Firth penalization and the additional sensitivity analyses yielded similar estimates. Adjusted PPC risks were 2.5% for robotic, 8.2% for laparoscopic, and 11.2% for open partial nephrectomy. Most PPCs were low grade. In radical nephrectomy, open surgery did not differ from laparoscopy; no inference was possible for the 18 robotic radical cases with no events. Robotic cases had longer intubation and higher airway pressures, but similar tidal volumes and oxygenation. Robotic partial nephrectomy was associated with lower adjusted odds of a 30-day PPC composite. The observational design does not establish a pulmonary mechanism or robotic superiority, and clinically significant events were sparse. Multicenter validation is required.

**Supplementary Information:**

The online version contains supplementary material available at 10.1007/s11701-026-03779-7.

## Introduction

Postoperative pulmonary complications (PPCs) are a major source of morbidity, prolonged hospitalization, and excess cost after surgery [1–3]. Renal surgery is anatomically distinctive because the operative field lies close to the diaphragm, pleura, and costodiaphragmatic recess. Pleural violation, incisional pain, impaired diaphragmatic excursion, and reduced cough effectiveness may therefore contribute to pulmonary morbidity after nephrectomy [4–6]. Partial nephrectomy is the preferred nephron-sparing strategy for clinically localized renal masses when feasible [7, 8], and minimally invasive platforms, particularly robot-assisted surgery, are widely used.

Recent work in heterogeneous abdominal and pelvic surgery reported higher early PPC rates after robotic than conventional laparoscopic surgery and attributed much of the signal to longer ventilation and pneumoperitoneum [9–12]. However, mixed cohorts combine procedures with different incisions, positions, pain profiles, and postoperative respiratory consequences. Associations observed across these heterogeneous procedures may not apply directly to nephrectomy, where the principal alternative to minimally invasive access is often an open flank or subcostal incision with its own respiratory burden [13, 14].

Comparative nephrectomy studies are also vulnerable to confounding by indication because open surgery is preferentially selected for larger or more complex tumors [15]. Moreover, prior renal-surgery studies have emphasized broad surgical outcomes rather than procedure-specific pulmonary events integrated with intraoperative ventilation data. We therefore examined the association between robotic, laparoscopic, and open access and 30-day PPCs after partial and radical nephrectomy. We prespecified procedure-stratified models to account for the different complexity structures of partial and radical nephrectomy and used additional analyses to evaluate sparse data, measured pulmonary confounders, postoperative imaging, and calendar-time selection.

## Materials and methods

### Study design and cohort

We conducted a retrospective cohort study of consecutive adults undergoing partial or radical nephrectomy by robotic, laparoscopic, or open access at a tertiary referral center from April 27, 2008, through October 5, 2025. Cases without a classified surgical approach or primary outcome, and procedures coded as neither partial nor radical nephrectomy, were excluded. The Sheba Medical Center institutional review board approved the study (SMC-23-4146) and waived individual informed consent. The study followed the STROBE reporting guideline.

### Exposure, access route, and conversion

Surgical approach was determined from the operative record and classified as robotic, laparoscopic, or open. Robotic and laparoscopic procedures were predominantly transperitoneal: among partial nephrectomies, 525 of 538 robotic cases (97.6%) and 166 of 167 laparoscopic cases (99.4%) used transperitoneal access. Conversion records identified 49 definite conversions to open surgery linked to the analytic cohort (22 initially robotic, 25 initially laparoscopic, and 2 minimally invasive cases with unspecified platform); 3 converted cases had a PPC. Earlier operative records did not consistently distinguish the initially intended approach from the completed approach. Conversions were therefore reported descriptively rather than reclassified in an adjusted as-treated model. Open partial nephrectomy generally used flank access, whereas open radical nephrectomy used flank, subcostal, or midline access according to tumor and surgeon factors.

### Outcome definition and ascertainment

The primary outcome was a prespecified 30-day PPC composite comprising atelectasis, pneumonia, pleural effusion, pneumothorax, pulmonary edema, or respiratory failure. Events were abstracted by structured review of clinical notes, discharge records, radiology reports, and complication fields. Reviewers were not blinded because surgical approach was evident in the record. Respiratory failure was classified from the clinician-documented postoperative diagnosis; objective escalation markers, including oxygen escalation, noninvasive support, reintubation, and PPC-related intensive care, were abstracted separately. Because a uniform retrospective physiologic threshold could not be applied across the full study period, this limitation is stated explicitly. Outcome definitions and severity grading followed published consensus and Clavien-Dindo frameworks [16, 17]. The primary composite retained all prespecified documented events, including radiographically detected events; therefore, potential surveillance bias was evaluated through imaging-frequency and severity analyses.

### Covariates and tumor complexity

Baseline variables included age, sex, body mass index (BMI), documented smoking history, Charlson Comorbidity Index, chronic pulmonary disease, and American Society of Anesthesiologists (ASA) physical status. Complexity adjustment was procedure specific. In partial nephrectomy, a complete R.E.N.A.L. nephrometry score and its component descriptors were available for all 1,008 cases after structured extraction supplemented by physician review of preoperative imaging records. Each nephrometry component was recorded separately and the total score was derived according to the original R.E.N.A.L. criteria [18]. The primary model included the complete R.E.N.A.L. score and tumor location. In radical nephrectomy, clinical tumor size served as the tumor-burden variable. Because pathologic stage and histology were incompletely documented in historical records, they were summarized descriptively rather than used as adjustment variables.

### Intraoperative physiologic data

Minute-level mean arterial pressure, heart rate, oxygen saturation, positive end-expiratory pressure (PEEP), peak inspiratory pressure (PIP), and tidal volume were extracted from the electronic anesthesia system. Complete ventilatory mechanics were available for the digital anesthesia subcohort (*n* = 1,059). Ventilation followed institutional lung-protective principles, with specific targets determined by the attending anesthesiologist [19, 20]. Plateau pressure, driving pressure, and dynamic compliance were not consistently available. Tidal volume indexed to ideal body weight was calculable in a linked subset and is reported descriptively in Online Resource 1. Ventilation variables were not entered into the primary outcome model because they were available only in a subcohort and may lie on the pathway between surgical approach and outcome.

### Statistical analysis

Categorical variables were compared with the chi-square or Fisher exact test and continuous variables with distribution-appropriate unequal-variance or nonparametric tests. The primary partial-nephrectomy logistic model included approach, complete R.E.N.A.L. score, tumor location, age, sex, Charlson Comorbidity Index, chronic pulmonary disease, and ASA physical status; laparoscopy was the reference. After expansion of categorical terms, the model contained 47 PPC events and 13 slope coefficients (3.6 events per coefficient) and converged without separation warnings. Given the low events-per-parameter ratio, Firth penalized logistic regression was performed as a sparse-data sensitivity analysis [21]. Additional partial-nephrectomy models added BMI (available for 953 cases), a binary indicator of any current or former smoking history documented in the medical record, chest radiography within 7 days, or calendar year. Absence of documented smoking history was not interpreted as confirmed never-smoking status. The chest-radiography model was considered an exploratory ascertainment analysis because imaging was measured after surgery and may reflect clinical suspicion. Adjusted predicted probabilities and marginal risk differences were obtained from the primary model with bootstrap confidence intervals. A clinically significant PPC analysis based on Clavien-Dindo grade II or higher was interpreted descriptively because events were sparse. A parallel radical-nephrectomy model substituted tumor size for nephrometry and compared open with laparoscopic surgery; robotic radical nephrectomy was descriptive because no PPCs occurred in 18 cases. Review of the propensity-score distributions, weights, and covariate balance showed inadequate overlap among approaches across calendar time. The originally submitted weighted estimate was therefore removed and was not used to support the conclusions [22]. All tests were two-sided, with *P*<.05 considered significant. Analyses used SPSS version 28.0. Supplemental Firth and bootstrap analyses were independently checked.

## Results

### Cohort characteristics

The cohort included 1,503 nephrectomies: 1,008 partial and 495 radical procedures. Median age was 63.9 years (IQR, 54.2–71.5), 1,007 patients (67.0%) were male, and 68 patients (4.5%) had at least 1 PPC. Surgical approach was robotic in 556 cases, laparoscopic in 444, and open in 503. Robotic cases were predominantly partial nephrectomies, whereas laparoscopic cases more often represented radical nephrectomy, supporting the procedure-stratified design. BMI was available for 1,426 patients and did not differ by approach. A positive current or former smoking history was documented in 129 patients; absence of such documentation was not interpreted as never-smoking. The documented-history indicator did not differ significantly among approaches (Table 1).

**Table 1:** Baseline characteristics by surgical approach

Characteristic	Overall (*N* = 1,503)	Robotic (*n* = 556)	Laparoscopic (*n* = 444)	Open (*n* = 503)	*P* value
**Age**,** median (IQR)**,** y**	63.9 (54.2–71.5)	65.6 (56.3–72.4)	62.6 (53.3–70.5)	63.3 (53.3–70.8)	0.002
**BMI**,** median (IQR)**,** kg/m2**	27.6 (24.8–31.2)	27.6 (24.8–31.0)	27.8 (24.8–31.6)	27.5 (24.7–31.1)	0.613
**Male sex**,** No. (%)**	1,007 (67.0)	379 (68.2)	297 (66.9)	331 (65.8)	0.68
**ASA physical status**,** median (IQR)**	2.0 (2.0–3.0)	2.0 (2.0–3.0)	2.0 (2.0–3.0)	2.0 (2.0–3.0)	0.14
**Charlson Comorbidity Index**,** median (IQR)**	3.0 (2.0–4.0)	3.0 (2.0–4.0)	2.0 (2.0–4.0)	3.0 (2.0–4.0)	0.09
**Chronic pulmonary disease**,** No. (%)**	99 (6.6)	42 (7.6)	21 (4.7)	36 (7.2)	0.15
**Diabetes mellitus**,** No. (%)**	279 (18.6)	86 (15.5)	91 (20.5)	102 (20.3)	0.07
**Positive current or former smoking history documented**,** No. (%)**	129 (8.6)	38 (6.8)	38 (8.6)	53 (10.5)	0.100
**Partial nephrectomy**,** No. (%)**	1,008 (67.1)	538 (96.8)	167 (37.6)	303 (60.2)	< 0.001
**Radical nephrectomy**,** No. (%)**	495 (32.9)	18 (3.2)	277 (62.4)	200 (39.8)	< 0.001
R.E.N.A.L. nephrometry score, median (IQR)a	7.0 (5.0–9.0)	7.0 (5.0–9.0)	7.0 (5.0–9.0)	8.0 (6.0–9.0)	0.62
**Clinical tumor size**,** median (IQR)**,** cmb**	3.7 (2.3–4.9)	3.2 (2.4–4.5)	3.4 (2.0-4.1)	4.1 (3.0-5.6)	< 0.001

ASA, American Society of Anesthesiologists; BMI, body mass index; IQR, interquartile range. BMI was available for 1,426 patients. Positive current or former smoking history was defined by affirmative documentation in the medical record. Absence of such documentation was not interpreted as confirmed never-smoking status. a R.E.N.A.L. nephrometry score was available for all 1,008 partial-nephrectomy cases after structured extraction supplemented by physician review of preoperative imaging records. b Tumor size was available for 1,471 cases

### Intraoperative physiologic profile

In the digital anesthesia subcohort, mean PIP was higher with robotic than laparoscopic or open surgery (27.1 vs. 21.5 and 16.1 cm H2O; *P*<.001), as was mean PEEP (5.8 vs. 4.9 and 4.2 cm H2O; *P*<.001). Mean tidal volume was similar (approximately 412 mL; *P*=.08), and oxygen saturation did not differ (*P*=.22). Intubation duration was longest in robotic cases (Table 2). Tidal volume indexed to ideal body weight was available in a limited linked subset (*n* = 644) and was reported descriptively because availability was highly unequal among platforms (Online Resource 1). These measurements describe exposure differences but do not establish a mechanism linking approach to PPCs.

**Table 2:** Intraoperative characteristics by surgical approach

Variable	Overall	Robotic	Laparoscopic	Open	*P* value
**Operative time**,** mean (SD)**,** min**	169 (68)	185 (68)	155 (55)	165 (72)	< 0.001
**Intubation duration**,** mean (SD)**,** min**	198 (76)	215 (75)	180 (62)	195 (80)	< 0.001
**Mean arterial pressure**,** mean (SD)**,** mm Hg**	89.0 (12.3)	89.2 (11.5)	88.5 (12.8)	89.0 (12.1)	0.72
**Peak inspiratory pressure**,** mean (SD)**,** cm H2O**	23.4 (14.5)	27.1 (12.9)	21.5 (15.7)	16.1 (15.4)	< 0.001
**PEEP**,** mean (SD)**,** cm H2O**	5.2 (2.0)	5.8 (2.1)	4.9 (1.8)	4.2 (1.5)	< 0.001
**Tidal volume**,** mean (SD)**,** mL**	412 (90)	415 (85)	405 (92)	420 (95)	0.08
**SpO2**,** mean (SD)**,** %**	98.0 (1.5)	98.1 (1.4)	97.8 (1.6)	97.9 (1.5)	0.22

PEEP, positive end-expiratory pressure; SD, standard deviation; SpO2, peripheral oxygen saturation. Operative and intubation times were available for the full cohort. Hemodynamic data were available for 1,460 patients. Ventilatory mechanics were available for 1,059 patients (robotic 530, laparoscopic 226, open 303)

### Postoperative outcomes

PPCs occurred in 19 of 556 robotic cases (3.4%), 18 of 444 laparoscopic cases (4.1%), and 31 of 503 open cases (6.2%; *P*=.09). In partial nephrectomy, PPC rates were 3.5% (19/538), 4.2% (7/167), and 6.9% (21/303), respectively (*P*=.08). In radical nephrectomy, PPCs occurred in 0 of 18 robotic cases, 11 of 277 laparoscopic cases (4.0%), and 10 of 200 open cases (5.0%; *P*=.53). Atelectasis was the most frequent component (Table 3). Chest radiography within 7 days was performed in 24.3% of robotic, 18.9% of laparoscopic, and 54.9% of open cases (*P*<.001), confirming differential surveillance intensity.

**Table 3:** Postoperative outcomes by surgical approach

Outcome	Overall (*N* = 1,503)	Robotic (*n* = 556)	Laparoscopic (*n* = 444)	Open (*n* = 503)	*P* value
**30-day PPC composite**,** No. (%)**	68 (4.5)	19 (3.4)	18 (4.1)	31 (6.2)	0.09
Partial nephrectomy	47/1,008 (4.7)	19/538 (3.5)	7/167 (4.2)	21/303 (6.9)	0.08
Radical nephrectomy	21/495 (4.2)	0/18 (0)	11/277 (4.0)	10/200 (5.0)	0.53
**Atelectasis**,** No. (%)**	34 (2.3)	7 (1.3)	7 (1.6)	20 (4.0)	0.01
**Pneumonia**,** No. (%)**	17 (1.1)	5 (0.9)	5 (1.1)	7 (1.4)	0.77
**Pleural effusion**,** No. (%)**	8 (0.5)	4 (0.7)	1 (0.2)	3 (0.6)	0.53
**Pneumothorax**,** No. (%)**	2 (0.1)	0 (0)	0 (0)	2 (0.4)	0.13
**Pulmonary edema**,** No. (%)**	6 (0.4)	2 (0.4)	3 (0.7)	1 (0.2)	0.55
**Respiratory failure**,** No. (%)**	6 (0.4)	1 (0.2)	2 (0.5)	3 (0.6)	0.55
**Length of stay**,** median (IQR)**,** d**	4.6 (3.5–6.6)	3.5 (2.6–4.6)	4.6 (3.5–6.6)	6.6 (5.5–8.5)	< 0.001
**Prolonged stay (> 6.6 d)**,** No. (%)**	369 (24.6)	46 (8.3)	100 (22.5)	223 (44.3)	< 0.001

PPC, postoperative pulmonary complication; IQR, interquartile range. Prolonged length of stay was prespecified as the cohort 75th percentile and is descriptive

### Adjusted and sensitivity analyses

**Fig. 1:**
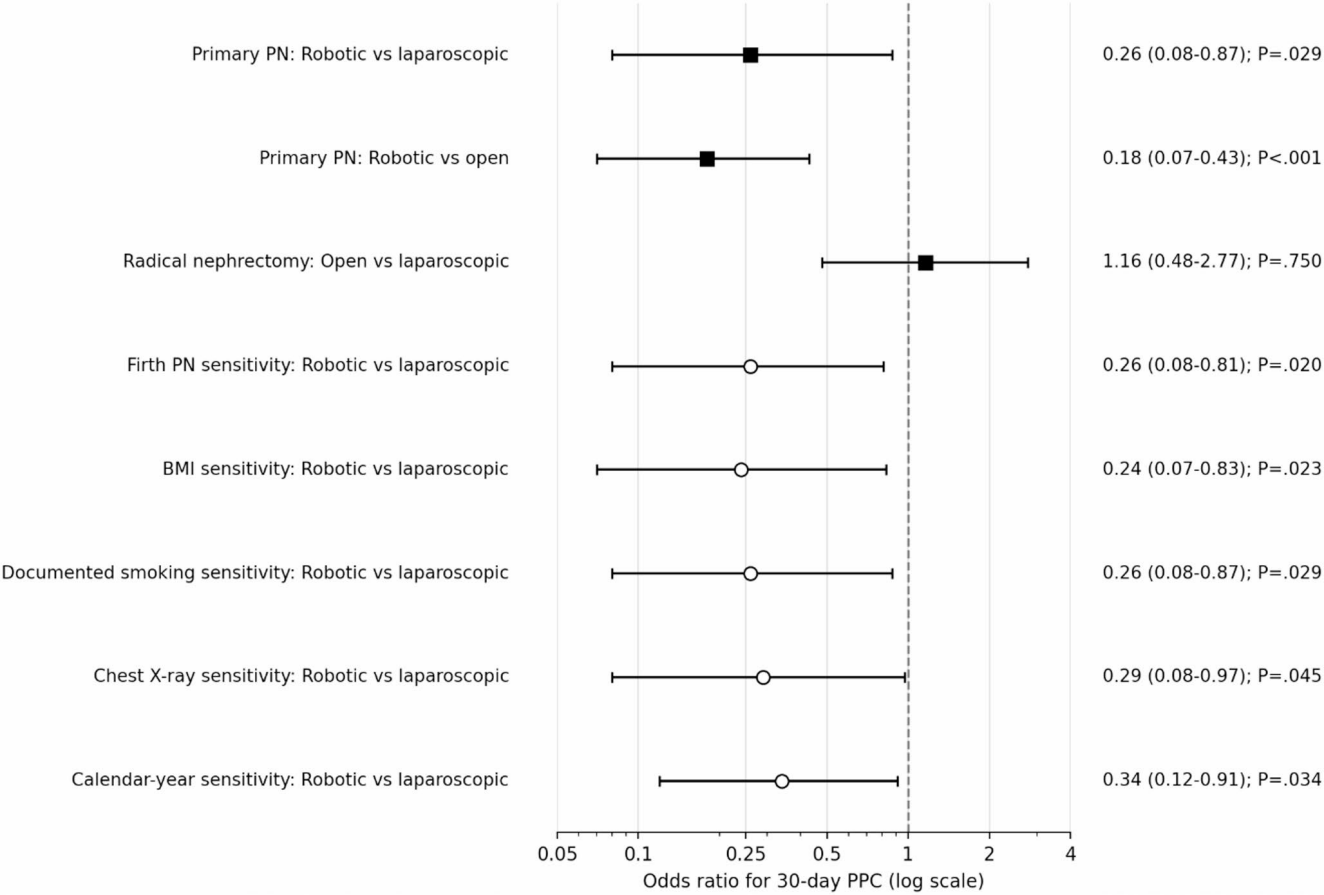
Procedure-stratified and sensitivity-model odds ratios for 30-day postoperative pulmonary complications. Primary partial-nephrectomy estimates were adjusted for complete R.E.N.A.L. score, tumor location, age, sex, Charlson Comorbidity Index, chronic pulmonary disease, and ASA physical status. The radical-nephrectomy model substituted tumor size. Sensitivity models used Firth penalization or added BMI, the documented current/former smoking-history indicator, week-1 chest radiography, or calendar year. Laparoscopy was the reference except for the robotic-versus-open contrast. Squares indicate primary models and circles indicate sensitivity models

In the primary partial-nephrectomy model, robotic surgery was associated with lower adjusted PPC odds than laparoscopy (adjusted odds ratio [aOR], 0.26; 95% CI, 0.08–0.87; *P*=.029) and open surgery (aOR, 0.18; 95% CI, 0.07–0.43; *P*<.001). Firth penalization yielded a similar robotic-versus-laparoscopic estimate (OR, 0.26; 95% CI, 0.08–0.81; *P*=.020). The estimate changed little after adding BMI (OR, 0.24; 95% CI, 0.07–0.83), the documented current/former smoking-history indicator (OR, 0.26; 95% CI, 0.08–0.87), or week-1 chest radiography (OR, 0.29; 95% CI, 0.08–0.97) (Table 4; Fig. 1). The chest-radiography analysis was exploratory because imaging occurred after surgery. A calendar-year model yielded a similar estimate (aOR, 0.34; 95% CI, 0.12–0.91), although residual era effects remain possible. The originally submitted IPTW estimate was removed after review of propensity-score distributions and covariate balance showed inadequate temporal overlap; it was not used to support the primary association.

**Table 4:** Procedure-stratified adjusted models and sensitivity analyses

Analysis and comparison	*N*	Events	OR	95% CI	*P* value
**Primary partial-nephrectomy model**					
**Robotic vs. laparoscopic**	1,008	47	0.26	0.08–0.87	0.029
**Robotic vs. open**	1,008	47	0.18	0.07–0.43	< 0.001
**Radical-nephrectomy model**					
**Open vs. laparoscopic**	477	21	1.16	0.48–2.77	0.75
**Partial-nephrectomy sensitivity analyses**					
**Firth: robotic vs. laparoscopic**	1,008	47	0.26	0.08–0.81	0.020
**Added BMI: robotic vs. laparoscopic**	953	47	0.24	0.07–0.83	0.023
**Added documented current/former smoking-history indicator: robotic vs. laparoscopic**	1,008	47	0.26	0.08–0.87	0.029
**Added week-1 chest X-ray: robotic vs. laparoscopic**	1,008	47	0.29	0.08–0.97	0.045
**Added calendar year: robotic vs. laparoscopic**	1,008	47	0.34	0.12–0.91	0.034

OR, odds ratio; BMI, body mass index. The primary partial-nephrectomy model included approach, complete R.E.N.A.L. score, tumor location, age, sex, Charlson Comorbidity Index, chronic pulmonary disease, and ASA physical status. The radical model substituted clinical tumor size for nephrometry and excluded robotic radical nephrectomy because no events occurred in 18 cases. Sensitivity models added the listed variable to the primary covariates. Firth regression addressed sparse-data bias

### Adjusted absolute risk and clinical severity

Adjusted partial-nephrectomy PPC probabilities were 2.5% (95% bootstrap CI, 1.5%-4.1%) for robotic, 8.2% (2.9%-16.7%) for laparoscopic, and 11.2% (5.8%-17.3%) for open surgery. The adjusted risk difference was − 5.7% points for robotic versus laparoscopic surgery (95% CI, -14.4 to 0.1) and − 8.7 points for robotic versus open surgery (95% CI, -15.2 to -2.6). During structured secondary review, 52 of 68 PPCs were classified as incidental or low-grade findings and 16 as symptomatic; 53 were Clavien-Dindo grade I and 15 were grade II or higher. Oxygen escalation occurred in 6 patients, reintubation in 2, and PPC-related ICU care in 5. A clinically significant endpoint analysis was underpowered and did not show a statistically significant difference by approach. These findings support cautious interpretation of the broad composite.

### Radical nephrectomy, conversion, and access route

In radical nephrectomy, open surgery did not differ significantly from laparoscopy (aOR, 1.16; 95% CI, 0.48–2.77; *P*=.75). No inferential conclusion can be drawn for robotic radical nephrectomy because that subgroup contained only 18 cases and no events. Malignancy was documented in 17 of 18 robotic, 222 of 277 laparoscopic, and 140 of 200 open radical cases; pathologic stage was incomplete, particularly in historical open cases (Online Resource 1). Among 49 linked definite conversions to open surgery, 3 PPCs occurred. Nearly all minimally invasive procedures were transperitoneal; retroperitoneal access accounted for 13 robotic and 1 laparoscopic partial nephrectomy. Detailed conversion, access-route, and pathology data are provided in Online Resource 1.

## Discussion

### Principal findings

In this procedure-stratified cohort, robotic partial nephrectomy was associated with lower adjusted odds of a broad 30-day PPC composite than laparoscopic or open partial nephrectomy. The Firth model and additional sensitivity analyses yielded similar estimates. Adjusted absolute risks suggested a potentially meaningful difference, although the robotic-versus-laparoscopic risk-difference interval narrowly included zero. These findings represent an observational association and do not establish that robotic surgery itself reduces pulmonary risk or explain the underlying mechanism.

### Interpretation of the pulmonary composite

The severity review is central to interpretation. Most PPCs were low-grade or incidental radiographic findings, and clinically significant events were uncommon. Open cases underwent substantially more early chest imaging, creating a plausible opportunity for surveillance bias. An exploratory model that included chest radiography did not eliminate the robotic-versus-laparoscopic association, but postoperative imaging is not a baseline confounder and this analysis cannot fully correct indication-dependent surveillance. Accordingly, the study supports an association for the prespecified broad composite, not a proven reduction in severe respiratory morbidity.

### Physiologic context

Robotic cases had longer intubation and higher PIP and PEEP, but similar tidal volumes and oxygenation. Higher airway pressure during pneumoperitoneum may reflect reduced chest-wall compliance rather than increased alveolar stress [9, 23]. Plateau pressure, driving pressure, and dynamic compliance were unavailable, and tidal volume indexed to ideal body weight was available only in a limited, unevenly distributed subset. Therefore, the ventilation data show that exposure patterns differed among approaches, but they cannot establish why PPC rates differed. Statements that robotics preserves postoperative respiratory mechanics or overcomes a ventilation penalty are not supported by these data.

### Access-related and procedure-specific factors

A procedure-specific explanation remains plausible but hypothesis-generating. Open flank or subcostal access may increase pain, splinting, and diaphragmatic limitation [13, 14], whereas minimally invasive access reduces incisional trauma. At the same time, robotic and laparoscopic procedures differ in selection, era, surgeon experience, operative route, and technical complexity. Nearly all minimally invasive cases in this cohort were transperitoneal, so the results should not be generalized to predominantly retroperitoneal programs. Conversion to open surgery was uncommon relative to the cohort but could alter postoperative respiratory recovery; historical coding prevented a fully reliable adjusted intended-versus-completed approach comparison.

### Relationship to prior literature

Meta-analyses of robotic versus laparoscopic partial nephrectomy have reported differences in warm ischemia, perioperative complications, and composite outcomes [24–26]. Prior institutional studies have also reported lower rates of selected complications after robotic partial nephrectomy [27, 28]. The present study extends this literature to pulmonary outcomes while addressing sparse data, measured pulmonary risk factors, imaging intensity, and absolute risk. It does not invalidate findings from mixed abdominal cohorts. Rather, associations reported across heterogeneous procedures may differ in nephrectomy and should be evaluated within the relevant operative and postoperative context.

### Limitations

This study has several limitations. First, its retrospective single-center design is vulnerable to confounding by indication, surgeon and team effects, changes in robotic adoption, enhanced-recovery pathways, analgesia, and postoperative care. Firth regression and the additional covariate analyses yielded similar estimates, but residual and unmeasured confounding remain possible. The originally submitted weighted estimate was removed because temporal overlap was inadequate, further emphasizing that the results should not be interpreted causally. Second, the primary partial-nephrectomy model had only 47 events and 13 slope coefficients; despite convergence, the low events-per-parameter ratio limits precision. Third, comprehensive never/former/current smoking status was unavailable. The exploratory smoking model used a binary indicator of any current or former smoking history documented in the medical record; absence of documentation did not establish never-smoking status. Fourth, early chest imaging differed substantially by approach, and the primary composite included surveillance-sensitive events. Respiratory failure was based on clinician documentation because a uniform retrospective physiologic threshold was unavailable. Fifth, conversion coding did not consistently distinguish intended and completed approach across the entire historical period. Sixth, the digital anesthesia and tidal-volume-per-ideal-body-weight subsets were incomplete, and driving pressure, dynamic compliance, pneumoperitoneum duration, obstructive sleep apnea, baseline oxygen requirement, and regional analgesia were unavailable or inconsistently recorded. Although R.E.N.A.L. data were complete after structured extraction and physician review, retrospective anatomical abstraction remains susceptible to measurement error. Finally, robotic radical nephrectomy included only 18 cases with no PPCs, precluding inference; pathologic stage was also incompletely documented in historical radical cases. Multicenter prospective validation with standardized pulmonary definitions and imaging practices is required.

## Conclusions

Among patients undergoing partial nephrectomy, robotic surgery was associated with lower adjusted odds of a broad 30-day PPC composite than laparoscopic or open surgery. Firth regression and the additional sensitivity analyses yielded similar estimates, but most events were low grade, clinically significant outcomes were sparse, and no respiratory mechanism can be inferred. Open and laparoscopic radical nephrectomy did not differ significantly, and the small zero-event robotic radical subgroup permits no inference. These findings warrant prospective multicenter validation before they influence approach selection.

## Supplementary Information

Below is the link to the electronic supplementary material.

Supplementary Material 1: Online Resource 1 contains model diagnostics, adjusted absolute risks, pulmonary-event severity, imaging use, conversion and access-route data, radical-nephrectomy pathology and stage summaries, ventilation indexed to ideal body weight, the calendar-time distribution of surgical approach.

## Data Availability

No datasets were generated or analysed during the current study.
