# Supplementary material for: Robotic, laparoscopic, and open nephrectomy and 30-day pulmonary morbidity: a procedure-stratified cohort study with intraoperative ventilation data
Source: J Robot Surg. 2026 Aug 11;20(1):818. doi: 10.1007/s11701-026-03779-7 (PMC13461887; doi:10.1007/s11701-026-03779-7)
Supplement: Supplementary file 1 [file 11701_2026_3779_MOESM1_ESM.pdf]

Online Resource 1

Robotic, Laparoscopic, and Open Nephrectomy and 30-Day Pulmonary Morbidity: A Procedure-Stratified Cohort Study With Intraoperative Ventilation Data

Husny Mahmud, MD, MPH; Amir Zabida, MD; Ido Amit, MD; Yoram Mor, MD; Haim Berkenstadt, MD; Dina Orkin, MD; Dorit E. Zilberman, MD; Barak Rosenzweig, MD; Orith Portnoy, MD; Menachem Laufer, MD; Zohar A. Dotan, MD, PhD

Journal of Robotic Surgery

Corresponding author: Husny Mahmud, MD, MPH; husny.mahmud@sheba.health.gov.il

This Online Resource contains supplementary methods, model diagnostics, sensitivity analyses, adjusted absolute risks, outcome-severity data, postoperative imaging, access route, conversion to open surgery, radical-nephrectomy pathology/stage, ventilation indexed to ideal body weight, and calendar-time distributions.

Supplementary Methods

Sparse-data and additional covariate sensitivity analyses

The primary partial-nephrectomy model contained 47 PPC events and 13 slope coefficients after expansion of categorical terms. Complete R.E.N.A.L. nephrometry scores and component descriptors were available for all 1,008 partial-nephrectomy cases after structured extraction supplemented by physician review of preoperative imaging records. Each component was recorded separately and the total score was derived according to the original R.E.N.A.L. criteria. The maximum-likelihood model and Firth penalized model both converged. BMI sensitivity used 953 cases with available BMI; a binary indicator identified any current or former smoking history documented in the medical record, and absence of documentation was not interpreted as confirmed never-smoking status; postoperative imaging sensitivity added any chest radiograph within 7 days and was treated as exploratory because imaging occurred after surgery. Adjusted predicted risks and risk differences were generated from the primary model with bootstrap confidence intervals.

Calendar-time overlap and removal of the IPTW analysis

The originally submitted IPTW estimate was reassessed during revision. The long study period showed marked temporal separation among approaches, and adequate common support could not be established. The weighted estimate was therefore removed from the revised manuscript and is not used to support the conclusions. Fig. S1 displays the calendar-time distribution of approach, and calendar year was examined in a separate outcome-model sensitivity analysis.

Severity review

All 68 PPC cases underwent a second structured review for symptoms, oxygen escalation, noninvasive support, reintubation, PPC-related ICU care, readmission, and Clavien-Dindo severity. The primary broad composite was retained as prespecified. Severity results are descriptive because clinically significant events were sparse.

Table S1 Model adequacy and sensitivity analyses for partial nephrectomy

Model	Comparison	N	Events	AUC	OR	95% CI	P value	Converged
**Primary PN adjusted logistic**	Robotic vs Laparoscopic	1008	47	0.774	0.260	0.077-0.872	.029	True
**Primary PN adjusted logistic**	Open vs Laparoscopic	1008	47	0.774	1.480	0.561-3.902	.428	True
**Primary PN Firth penalized sensitivity**	Robotic vs Laparoscopic	1008	47	0.773	0.258	0.082-0.811	.020	True

Model	Comparison	N	Events	AUC	OR	95% CI	P value	Converged
**Primary PN Firth penalized sensitivity**	Open vs Laparoscopic	1008	47	0.773	1.409	0.566-3.503	.461	True
**PN + BMI sensitivity**	Robotic vs Laparoscopic	953	47	0.766	0.245	0.072-0.826	.023	True
**PN + BMI sensitivity**	Open vs Laparoscopic	953	47	0.766	1.398	0.529-3.695	.499	True
**PN + documented current/former smoking-history indicator**	Robotic vs Laparoscopic	1008	47	0.774	0.260	0.077-0.872	.029	True
**PN + documented current/former smoking-history indicator**	Open vs Laparoscopic	1008	47	0.774	1.477	0.559-3.904	.431	True
**PN + chest X-ray 7d sensitivity**	Robotic vs Laparoscopic	1008	47	0.806	0.285	0.084-0.971	.045	True
**PN + chest X-ray 7d sensitivity**	Open vs Laparoscopic	1008	47	0.806	0.995	0.353-2.807	.992	True
**Hard-PPC Firth sensitivity**	Robotic vs Laparoscopic	1008	15	0.905	0.524	0.083-3.319	.493	True
**Hard-PPC Firth sensitivity**	Open vs Laparoscopic	1008	15	0.905	0.674	0.165-2.758	.583	True

The clinically significant PPC model was considered underpowered; detailed clinical-severity counts are provided in Tables S3-S4 rather than used to support an approach effect.

Table S2 Primary partial-nephrectomy model adequacy

Parameter count reflects all slope coefficients after expansion of categorical terms. The intercept was excluded from the events-per-parameter denominator. Complete R.E.N.A.L. score and tumor location were entered directly in the model.

Item	Value
**Analysis N**	1,008
**PPC events**	47
**Slope parameters**	13

Item	Value
**Intercept**	1
**Events per slope parameter**	3.62
**Events per parameter including intercept**	3.36
**Maximum-likelihood convergence**	Yes
**Firth convergence**	Yes

Table S3 Adjusted predicted 30-day PPC risks and marginal risk differences in partial nephrectomy

Approach	Adjusted risk, %	95% bootstrap CI, %
**Robotic**	2.5	1.5-4.1
**Laparoscopic**	8.2	2.9-16.7
**Open**	11.2	5.8-17.3
**Contrast**	**Risk difference, percentage points**	**95% bootstrap CI**
**Robotic vs Laparoscopic**	-5.7	-14.4 to 0.1
**Robotic vs Open**	-8.7	-15.2 to -2.6
**Open vs Laparoscopic**	3.0	-4.2 to 10.3

Table S4 Pulmonary-complication severity by surgical approach

Approach	PPC events	Symptomatic	Incidental/ low-grade	Oxygen	NIV/HFNC	Reintubation	PPC-related ICU	Readmission	Clavien >=II
**Robotic**	19	6	13	2	1	1	1	1	6
**Laparoscopic**	18	3	15	1	0	1	1	0	3
**Open**	31	7	24	3	0	0	3	0	6
**Overall**	**68**	**16**	**52**	**6**	**1**	**2**	**5**	**1**	**15**

NIV/HFNC, noninvasive ventilation or high-flow nasal cannula. Incidental/low-grade denotes events without documented symptoms or escalation in the structured severity review. Clavien grade II or higher was considered clinically significant for descriptive reporting.

Table S5 Pulmonary-complication severity by procedure stratum and approach

Procedure	Approach	PPC events	Symptomatic	Incidental/ low-grade	Oxygen	Reintubation	ICU	Clavien >=II
**Partial**	**Robotic**	19	6	13	2	1	1	6
**Partial**	**Laparoscopic**	7	1	6	0	0	0	1

Procedure	Approach	PPC events	Symptomatic	Incidental/ low-grade	Oxygen	Reintubation	ICU	Clavien >=II
**Partial**	**Open**	21	4	17	0	0	0	1
**Radical**	**Laparoscopic**	11	2	9	1	1	1	2
**Radical**	**Open**	10	3	7	3	0	3	5

Table S6 Week-1 chest radiography and minimally invasive access route

Measure	Robotic	Laparoscopic	Open	P value
**Chest radiograph within 7 d, No. (%)**	135/556 (24.3)	84/444 (18.9)	276/503 (54.9)	<.001
**Partial nephrectomy, transperitoneal, No. (%)**	525/538 (97.6)	166/167 (99.4)	Not applicable	
**Partial nephrectomy, retroperitoneal, No. (%)**	13/538 (2.4)	1/167 (0.6)	Not applicable	
**Radical nephrectomy, transperitoneal, No. (%)**	18/18 (100)	277/277 (100)	Not applicable	
**Radical nephrectomy, retroperitoneal, No. (%)**	0	0	Not applicable	

Chest-radiograph use differed by approach. Adding chest radiography to the partial-nephrectomy model yielded an OR of 0.285 (95% CI, 0.084-0.971; P=.045) for robotic versus laparoscopic surgery. Access-route data indicate that the minimally invasive findings predominantly apply to transperitoneal nephrectomy.

Table S7 Definite conversion to open surgery

Measure	No.
**Linked definite conversions**	49
**Initially robotic**	22
**Initially laparoscopic**	25
**Minimally invasive, platform unspecified**	2
**PPC among converted cases**	3

Earlier operative records did not consistently distinguish the initially intended approach from the completed approach. Consequently, conversions are reported descriptively, and no adjusted as-treated model was claimed.

Table S8 Radical-nephrectomy disease classification and pathologic stage

Measure	Robotic (n=18)	Laparoscopic (n=277)	Open (n=200)
**Benign, No. (%)**	1 (5.6)	13 (4.7)	7 (3.5)
**Malignant, No. (%)**	17 (94.4)	222 (80.1)	140 (70.0)
**Unknown benign/malignant status, No. (%)**	0	42 (15.2)	53 (26.5)

Measure	Robotic (n=18)	Laparoscopic (n=277)	Open (n=200)
**Pathologic stage documented, No. (%)**	17 (94.4)	195 (70.4)	95 (47.5)
**pT1a-pT1b, No. (%)**	11 (61.1)	83 (30.0)	14 (7.0)
**pT2/2a/2b, No. (%)**	2 (11.1)	36 (13.0)	11 (5.5)
**pT3/3a/3b/3c, No. (%)**	4 (22.2)	70 (25.3)	67 (33.5)
**pT4, No. (%)**	0	1 (0.4)	3 (1.5)
Other documented pathologic stage, No. (%)	0	5 (1.8)	0

Stage categories are pathologic, not clinical. Missing historical stage and pathology data were not imputed and precluded adjusted stage-stratified inference.

Table S9 Tidal volume indexed to ideal body weight in the linked subset

Approach	Available n	Mean (SD), mL/kg IBW	Median (IQR), mL/kg IBW
**Robotic**	486	6.33 (1.68)	6.08 (4.98-7.49)
**Laparoscopic**	29	5.57 (1.70)	5.13 (4.15-7.07)
**Open**	129	6.47 (1.80)	6.30 (5.04-7.69)

TV/IBW was available in 644 linked cases and was highly uneven by approach; therefore, these data are descriptive and should not be used for inferential comparisons among approaches. Plateau pressure, driving pressure, and dynamic compliance were not consistently available.

Fig. S1 Distribution of nephrectomy approach over calendar time

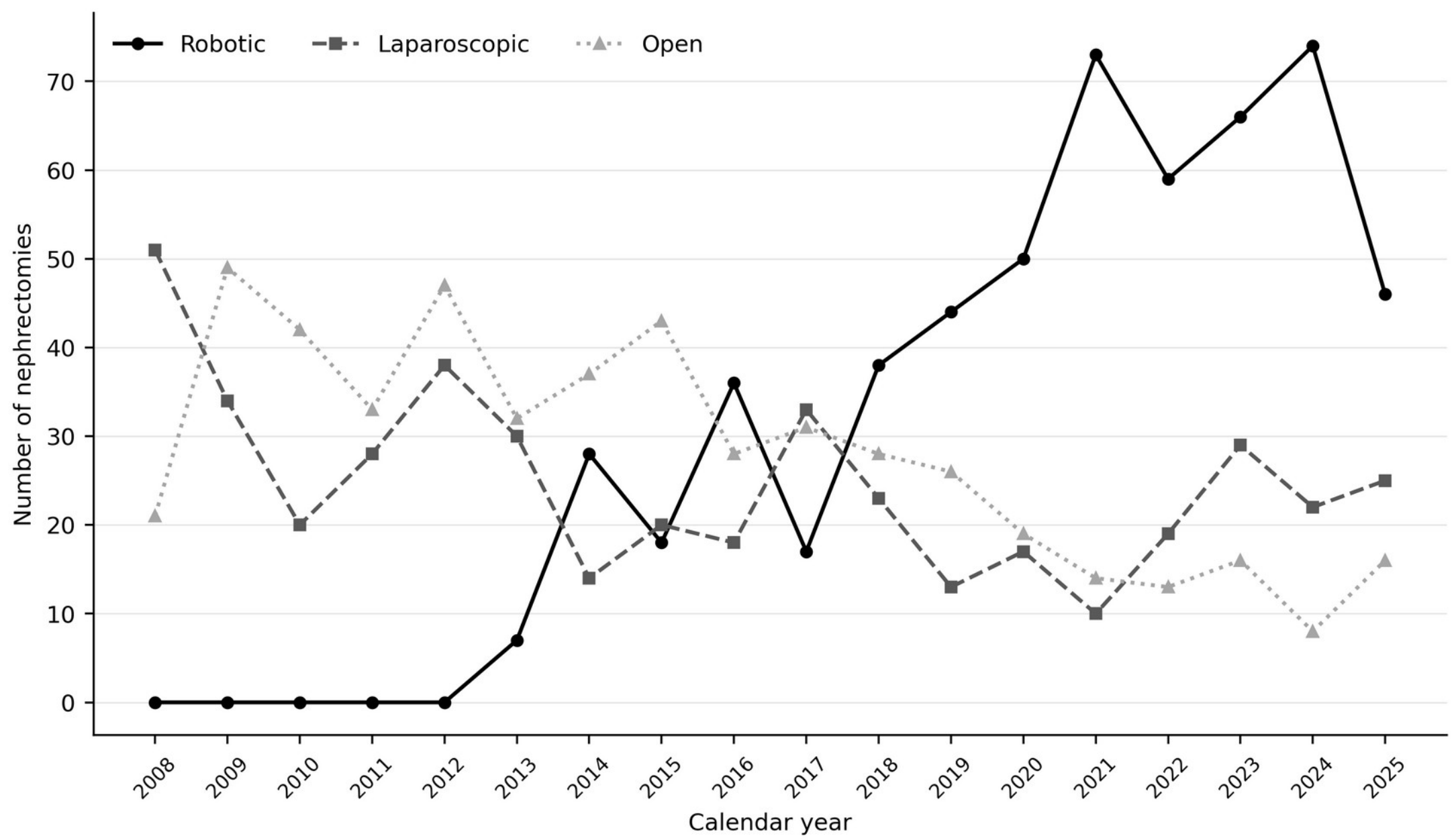

The marked temporal shift toward robotic surgery and away from conventional laparoscopy illustrates the potential for residual era effects. Calendar year was therefore evaluated in a sensitivity model and remained a limitation of the observational design.
